# Perirenal Fat CT Radiomics-Based Survival Model for Upper Tract Urothelial Carcinoma: Integrating Texture Features with Clinical Predictors

**DOI:** 10.3390/cancers16223772

**Published:** 2024-11-08

**Authors:** Abdulrahman Al Mopti, Abdulsalam Alqahtani, Ali H. D. Alshehri, Chunhui Li, Ghulam Nabi

**Affiliations:** 1 Centre for Medical Engineering and Technology, School of Medicine, University of Dundee, Dundee DD1 9SY, UK; axalqahtani@dundee.ac.uk (A.A.); g.nabi@dundee.ac.uk (G.N.); 2 Radiology Department, College of Applied Medical Sciences, Najran University, Najran 55461, Saudi Arabia; ahzafer@nu.edu.sa; 3 School of Science and Engineering, University of Dundee, Dundee DD1 4HN, UK; c.li@dundee.ac.uk

**Keywords:** upper tract urothelial carcinoma, perirenal fat radiomics, prognostic modeling, CT imaging, machine learning, texture analysis, survival, prognosis

## Abstract

**Background:** Upper tract urothelial carcinoma (UTUC) presents significant challenges in prognostication due to its rarity and complex anatomy. This study introduces a novel approach integrating perirenal fat (PRF) radiomics with clinical factors to enhance prognostic accuracy in UTUC. **Methods:** The study retrospectively analyzed 103 UTUC patients who underwent radical nephroureterectomy. PRF radiomics features were extracted from preoperative CT scans using a semi-automated segmentation method. Three prognostic models were developed: clinical, radiomics, and combined. Model performance was assessed using concordance index (C-index), time-dependent Area Under the Curve (AUC), and integrated Brier score. **Results**: The combined model demonstrated superior performance (C-index: 0.784, 95% CI: 0.707–0.861) compared to the radiomics (0.759, 95% CI: 0.678–0.840) and clinical (0.653, 95% CI: 0.547–0.759) models. Time-dependent AUC analysis revealed the radiomics model’s particular strength in short-term prognosis (12-month AUC: 0.9281), while the combined model excelled in long-term predictions (60-month AUC: 0.8403). Key PRF radiomics features showed stronger prognostic value than traditional clinical factors. **Conclusions:** Integration of PRF radiomics with clinical data significantly improves prognostic accuracy in UTUC. This approach offers a more nuanced analysis of the tumor microenvironment, potentially capturing early signs of tumor invasion not visible through conventional imaging. The semi-automated PRF segmentation method presents advantages in reproducibility and ease of use, facilitating potential clinical implementation.

## Simple Summary

Upper tract urothelial carcinoma (UTUC) is a rare but aggressive cancer that is difficult to predict and treat effectively. Current methods for assessing a patient’s outlook often fall short. Our research introduces a new approach that could change this. We look at the fat surrounding the kidney using detailed CT scan analysis, combined with standard clinical information. This method provides a more accurate prediction of how patients with UTUC might fare. By examining the texture and patterns in this fat tissue, we can potentially detect early signs of cancer spread that are not visible to the naked eye. Our findings could help doctors make better treatment decisions, such as whether to use chemotherapy before surgery or how extensive the surgery should be. This personalized approach aims to improve outcomes for patients with this challenging cancer.

## 1. Introduction

Upper tract urothelial carcinoma (UTUC) is a rare but aggressive malignancy, accounting for 5–10% of all urothelial carcinomas [1]. Although UTUC is relatively uncommon, it exhibits an aggressive nature with a significant tendency for both recurrence and disease advancement. Radical nephroureterectomy (RNU), which involves the surgical removal of the kidney, ureter, and a cuff of the bladder, is the standard treatment for high-risk UTUC [2,3]. Patients diagnosed with late-stage UTUC face a particularly grim prognosis, with less than half surviving beyond five years post-diagnosis [4,5]. The complex anatomy of the upper urinary tract and limitations of current diagnostic tools make early detection, accurate staging, and prognostication of UTUC particularly challenging, often leading to suboptimal treatment decisions and poor patient outcomes [6].

Prognostication in UTUC remains a significant challenge for clinicians. Current prognostic models rely heavily on postoperative pathological factors, which are not available preoperatively, limiting their utility in treatment planning [7]. Preoperative prognostic factors, including imaging findings and urinary biomarkers, have shown promise but lack the accuracy needed for confident clinical decision-making [8].

In recent years, the role of perirenal fat (PRF) in UTUC has gained attention as a potential prognostic factor. PRF stranding (PRFS), defined as linear areas of soft-tissue attenuation in the perirenal space, has emerged as a valuable prognostic indicator. Yanagi et al. demonstrated that high PRFS was associated with significantly lower progression-free survival rates compared to low PRFS in patients with renal pelvic urothelial carcinoma without hydronephrosis [9]. Similarly, Chung et al. found that PRFS was an independent prognostic factor for recurrence-free survival and cancer-specific survival in patients with ureteral urothelial carcinoma [10].

The current TNM staging system for UTUC includes PRF invasion as a criterion for T3a stage, emphasizing its prognostic significance [1]. However, the assessment of PRF involvement remains largely subjective and qualitative. Radiologists typically rely on visual inspection of computed tomography (CT) or magnetic resonance imaging (MRI) images, an approach subject to inter-observer variability that may miss subtle changes in fat composition or texture [9,10].

In addition, the quantity of PRF may also impact surgical outcomes. Yanagi et al. found that thick posterior PRF thickness was a preoperative risk factor for prolonged pneumoretroperitoneum time during retroperitoneal laparoscopic nephroureterectomy [11]. This finding highlights the importance of considering PRF characteristics in surgical planning and potentially in prognostication.

Despite growing recognition of perirenal fat’s importance in UTUC, current assessment methods remain largely subjective and qualitative. Radiologists typically rely on visual inspection of CT or MRI images, an approach subject to inter-observer variability that may miss subtle changes in fat composition or texture [12]. The Mayo Adhesive Probability (MAP) score, used primarily to predict surgical complexity due to perinephric fat adhesions in renal surgeries, highlights the clinical relevance of perirenal fat characteristics [13]. However, while the MAP score focuses on surgical outcomes in renal cancers, it does not provide a quantitative assessment of PRF texture for prognostication in UTUC.

The lack of objective, quantitative methods for analyzing PRF texture represents a significant gap in UTUC management and prognostication. Radiomics approaches have shown great promise in the analysis of tumor characteristics in various cancers, offering non-invasive insights into tumor heterogeneity and treatment response prediction [14,15,16]. Unlike previous studies that focused primarily on tumor-based radiomics, this study emphasized the prognostic value of perirenal fat characteristics. By analyzing PRF radiomics, a novel perspective on the tumor microenvironment’s role in UTUC progression was provided, which had been largely unexplored in prior research.

This study proposed a novel approach to UTUC prognostication by applying radiomics analysis to perirenal fat. By extracting quantitative features from CT images of PRF and integrating them with clinical factors, the study aimed to develop a more accurate and objective method for risk stratification in UTUC. This approach had the potential to overcome the limitations of current subjective assessments, enhance UTUC staging accuracy, improve risk stratification, and guide treatment decisions, ultimately providing clinicians with a potential tool for personalized UTUC management and improved prognostic accuracy.

## 2. Materials and Methods

### 2.1. Study Design and Patient Cohort

This retrospective study adhered to the Standards for Reporting Diagnostic Accuracy Studies (STARD) guidelines and the Checklist for Evaluation of Radiomics (CLEAR) [17]. The study analyzed computed tomography urography (CTU) scans and clinicopathological data of 103 patients with UTUC who underwent RNU between January 2000 and December 2022. Data were accessed from the Tayside Urological Cancers database under approval from the East of Scotland Research Ethical Service (Approval No. IGTCAL12931). The requirement for informed consent was waived under Caldicott Approval.

Inclusion criteria encompassed availability of CTU datasets adhering to a set protocol, histologically validated UTUC, and absence of prior endoscopic management for UTUC before CT assessment. Exclusion criteria resulted in the removal of patients due to non-contrast CT scans, poor-quality images, treatments prior to CT scans, missing clinical/pathology data, or inaccurate PRF segmentation.

### 2.2. CT Imaging Protocol

CT examinations were performed using a standardized protocol on a 64-slice multidetector CT scanner (Somatom Definition AS, Siemens Healthineers, Erlangen, Germany). The imaging protocol included non-contrast, nephrographic (100 s post-contrast), and excretory phase (10 min post-contrast) acquisitions. Contrast medium (100 mL of Omnipaque 300) was administered intravenously at a rate of 3 mL/s. Image reconstruction parameters included a slice thickness of 1 mm with 0.7 mm overlap.

### 2.3. Patient Follow-Up

Post-operative follow-up was conducted at regular intervals: every 3–4 months in the first year, every 6 months in the second and third years, and annually thereafter. The follow-up protocol included cystoscopy, routine blood tests, urinary cytology analyses, and chest and abdominal radiographic imaging. The primary endpoints were overall survival (OS) and recurrence rate.

### 2.4. Image Segmentation and Radiomics Feature Extraction

Three-dimensional segmentation of the PRF was performed using a semi-automated morphological approach in 3D Slicer (version 5.2.2), followed by an automated refinement process using Python 3.7. The segmentation extended from the tumor region of interest (ROI) up to 20 mm into the surrounding fat tissue, with a Hounsfield Unit (HU) threshold of −130 to −90 applied to specifically capture perirenal fat. A visual representation of CT images and segmentations are included in (Supplementary Figures S1 and S2).

Radiomics features were extracted using the PyRadiomics library, yielding a comprehensive set of 1409 features. These features encompassed first-order statistics, texture-based features derived from various matrices, and shape-based features. Image pre-processing included resampling to a 1 × 1 × 1 mm^3^ voxel size and discretization with a fixed bin width of 25 Hounsfield Units. Features were computed from original, filtered, and transformed (e.g., wavelet and exponential) images to capture multi-scale and multi-frequency texture information.

### 2.5. Feature Selection and Model Development

A multi-step process was employed for feature selection, starting with correlation filtering to remove redundant features with a correlation coefficient greater than 0.9. Stability analysis was then performed, retaining only features with an intraclass correlation coefficient greater than 0.7. Univariate analysis followed to identify features significantly associated with the outcomes, and significant features were included in a multivariate analysis. Least absolute shrinkage and selection operator (LASSO) regression was applied as the final step to select the most robust features. Three Cox proportional hazards models were developed: a clinical model incorporating established prognostic factors, a radiomics model using the selected radiomics features, and a combined model integrating both clinical and radiomics features.

Furthermore, a comparison was conducted to evaluate the best model’s performance with the addition of the radiomics signature of the tumor from the most recent published work [18].

### 2.6. Statistical Analysis and Model Evaluation

Model performance was assessed using multiple metrics, including the concordance index (C-index), time-dependent Area Under the Curve (AUC), and Kaplan–Meier (KM) survival analysis. Hazard ratios (HR) were calculated for each variable in the models to quantify their impact on survival outcomes. The proportional hazards assumption was tested for each model. Bootstrapping techniques were employed to generate C-index distributions and confidence intervals, assessing model stability and accuracy.

Comparative analysis of the models was conducted using AUC calculations and ROC curve visualizations at different time points (6, 12, 24, 36, and 60 months) to evaluate their discriminative power over time. Akaike Information Criterion (AIC) and Bayesian Information Criterion (BIC) were used to assess model complexity. All statistical analyses were performed using R statistical software (version 3.3.3), with a significance threshold set at *p* < 0.05.

## 3. Results

The study cohort comprised 103 patients diagnosed with UTUC. The median age was 74 years (range: 49–93), with a slight male predominance (58%). A history of smoking was common, with 76% of patients being current or former smokers. Tumor characteristics varied, with 70% classified as high-grade and 26% at stage T3 or T4. During the follow-up period, 54% of patients died, and 29% experienced disease recurrence (Table 1).

Univariate Cox regression analysis was conducted on both clinical variables and radiomics features to identify potential prognostic factors. In the univariate analysis, none of the clinical variables reached statistical significance at the *p* < 0.05 level. However, tumor size, hydronephrosis, stage, and smoking status showed trends that may warrant further investigation in multivariate models (Table 2). In contrast, numerous radiomics features demonstrated significant associations with survival outcomes (Table 3).

**Figure 1:**
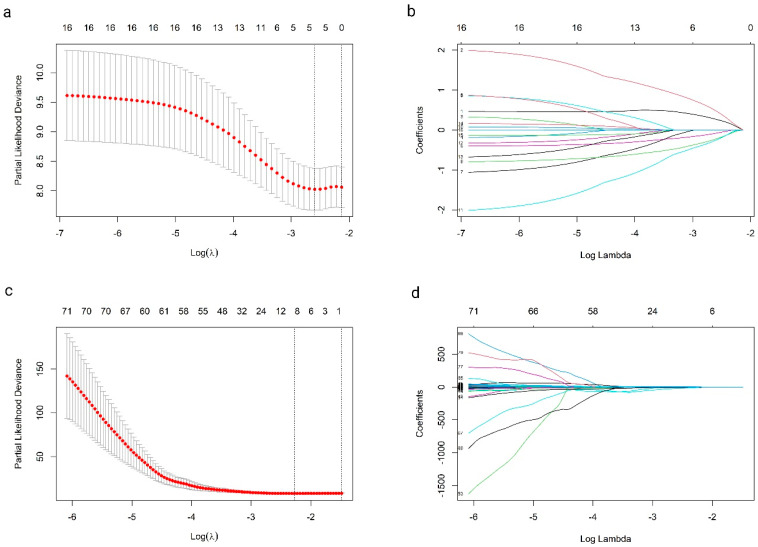
LASSO regression results for clinical (**a**,**b**) and radiomics models (**c**,**d**). (**a**,**c**) show partial likelihood deviance and optimal lambda selection, while (**b**,**d**) display the shrinking feature coefficients as lambda increases.

Before proceeding with the multivariate Cox regression analysis, a LASSO regression was applied to refine feature selection and identify the most robust predictors of survival outcomes. Initially, 16 clinical variables and 71 radiomics features were considered. The optimal lambda, chosen to minimize the partial likelihood deviance, reduced the number of features to six clinical variables and six radiomics features (Figure 1a–d). However, to avoid overfitting and ensure model stability, a further refinement step was carried out for the radiomics features. This involved assessing feature stability through cross-validation and removing any features prone to instability across different subsets of data. This step reduced the number of radiomics features to three features, as only the most stable and relevant features were retained.

The final feature selection, after the stability assessment and univariate analysis, is reflected in (Table 4), which presents the features that were ultimately used in the multivariate Cox regression models.

The multivariate analysis revealed distinct patterns across the three models. In the clinical model, only hydronephrosis and stage reached statistical significance (HR = 0.45, 95% CI: 0.20–0.98, *p* = 0.04) and (HR = 0.38, 95% CI: 0.16–0.88, *p* = 0.02), respectively. The radiomics model identified three independent prognostic factors, with wavelet.LHH_gldm_DependenceEntropy and exponential_glszm_GrayLevelNonUniformity significantly associated with better survival outcomes (HR = 0.40, 95% CI: 0.26–0.61, *p* < 0.001) and (HR = 0.49, 95% CI: 0.33–0.71, *p* < 0.001), while exponential_gldm_LargeDependenceHighGrayLevelEmphasis was linked to poorer outcomes (HR = 1.57, 95% CI: 1.12–2.22, *p* = 0.01). To aid in the interpretation of the radiomics features, detailed definitions are provided in Supplementary Table S1.

In the combined model, stage and hydronephrosis emerged as significant clinical factors (*p* = 0.03 and *p* = 0.01, respectively), while the three radiomics features remained highly significant (*p* < 0.01). The radiomics features demonstrated stronger statistical significance compared to clinical variables in both their respective models and the combined model. Several evaluation metrics, including the C-index, time-dependent AUC, integrated Brier score, and internal validation using bootstrap resampling, were used to assess and compare the predictive performance of the models. These performance metrics are detailed in (Table 5).

**Figure 2:**
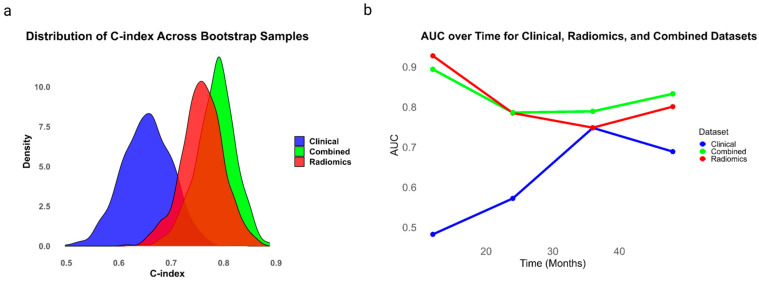
Includes two subfigures illustrating C-index and AUC over time for the models, represented by distinct colors. (**a**) uses the distribution of C-index as kernel density estimation for the radiomics, combined and clinical models, and (**b**) depicts the AUC over time for the models.

The multivariate analysis shows that the combined model consistently outperforms the clinical and radiomics models in predicting patient outcomes for UTUC. Specifically, the clinical model has a C-index of 0.65 (95% CI: 0.55–0.76), the radiomics model has a higher C-index of 0.76 (95% CI: 0.68–0.84), and the combined model achieves the highest C-index of 0.78 (95% CI: 0.71–0.86), indicating superior predictive accuracy. A graph plotting AUC over time for the three models shows the clinical model rising from 0.5 to 0.6 between months 20 and 40, while both the radiomics and combined models begin with higher AUCs of approximately 0.8, maintaining stability before the combined model slightly outperforms the radiomics model after months 40. These results are visually depicted in Figure 2, which includes the distribution of C-index as a kernel density estimation for the radiomics, combined and clinical models (Figure 2a) and AUC over time (Figure 2b).

**Figure 3:**
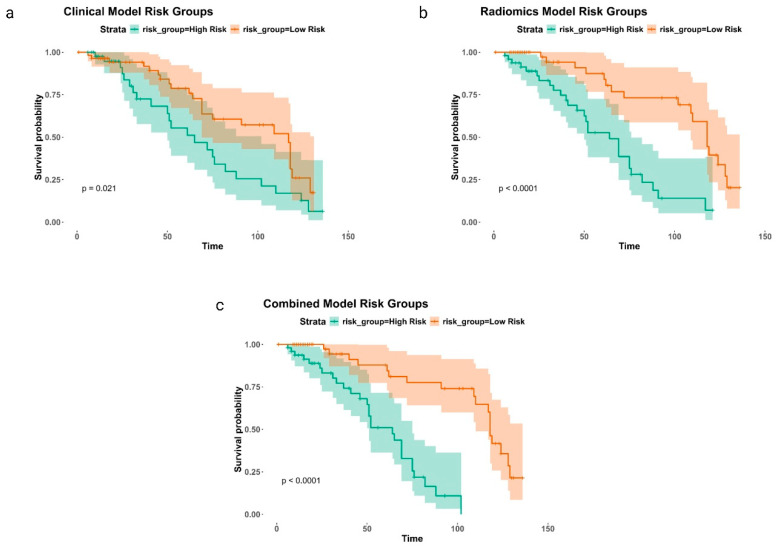
Presents three KM plots showing survival probabilities for the clinical, radiomics, and combined models, each stratifying cases into high-risk and low-risk groups. Panel (**a**) focuses on the clinical model, panel (**b**) on the radiomics model, and panel (**c**) on the combined model.

KM plots serve as model evaluations, demonstrating survival probabilities over time for the clinical, radiomics, and combined models, highlighting distinctions between high-risk and low-risk groups (Figure 3). The KM analysis revealed statistically significant differences between the groups for all models, with (*p* = 0.02) for the clinical model and (*p* < 0.0001) for both the radiomics and combined models.

**Figure 4:**
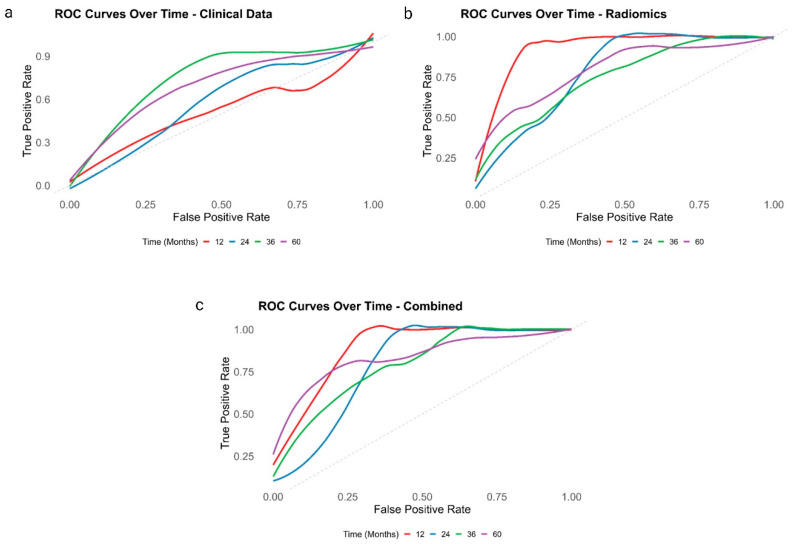
Comprises three subfigures illustrating ROC curves for different models evaluated at various time points, each represented by a distinct color. Panel (**a**) focuses on the clinical model, panel (**b**) on the radiomics model, and panel (**c**) on the combined model, highlighting their discriminative abilities.

ROC plots analyzed depict the evolving performance of clinical, radiomics, and combined models over time, demonstrating each model’s capacity to accurately discriminate between diagnostic groups. At 12 months, the radiomics model showed the highest AUC of 0.93, slightly outperforming the combined model’s AUC of 0.89 and significantly surpassing the clinical model’s AUC of 0.53. However, at 36 and 60 months, the combined model demonstrated superior predictive performance, achieving AUCs of 0.79 and 0.84, respectively. This was higher compared to the clinical model’s AUCs of 0.75 and 0.69 and the radiomics model’s AUCs of 0.75 and 0.80 at the same time points. These results suggest the combined model’s superior predictive power across multiple time points (Figure 4).

**Figure 5:**
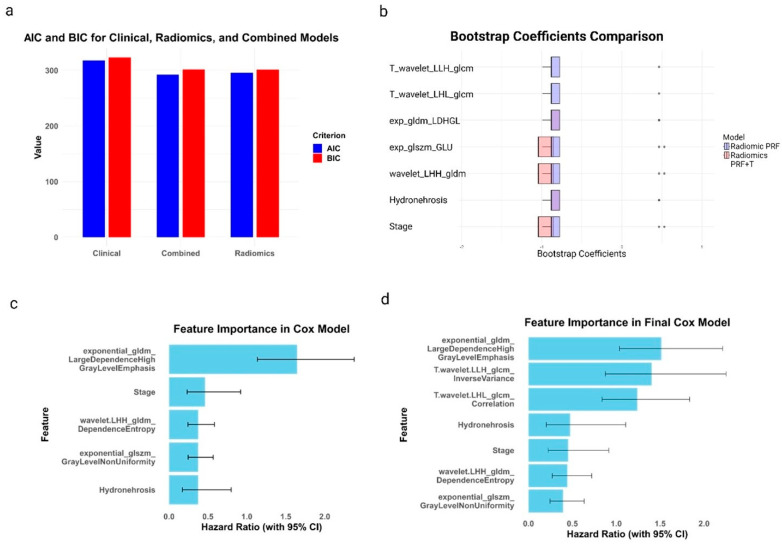
Model performance and feature importance in predicting UTUC outcomes. (**a**) AIC and BIC values for clinical, radiomics, and combined models. (**b**) Bootstrap coefficients comparison between radiomics and combined models; grey dots represent individual bootstrap samples, indicating the variability and stability of each feature’s coefficient across samples. (**c**) Feature importance in the Cox model combining PRF radiomics and clinical variables. (**d**) Feature importance in the final Cox model incorporating PRF radiomics, tumor radiomics, and clinical variables. HRs with 95% CIs are shown.

The combined model incorporating both clinical and radiomics features demonstrated superior performance, as evidenced by the lowest AIC and BIC values. Specifically, the combined model had an AIC of 292.53 and a BIC of 301.68, which are lower than those of the radiomics model (AIC: 296.03, BIC: 301.51) and the clinical model (AIC: 318.12, BIC: 323.61) (Figure 5a). Bootstrap coefficient analysis revealed the stability and complementary nature of tumor-specific, PRF radiomics, and clinical features in the combined model (Figure 5b). Feature importance analysis highlighted the strong predictive power of the exponential_gldm_LargeDependenceHighGrayLevelEmphasis feature from PRF in both the initial combined model (Figure 5c) and the final model that included tumor radiomics (Figure 5d). The introduction of tumor-specific radiomics features (T.wavelet.LLH_glcm_InverseVariance and T.wavelet.LHL_glcm_Correlation) in the final model demonstrated their added value in UTUC outcome prediction, while clinical variables (Hydronephrosis and Stage) remained important predictors across all models.

## 4. Discussion

The study introduces an approach to prognostic modeling in UTUC by integrating radiomics features derived from PRF with traditional clinical factors. This method demonstrates superior predictive performance compared to conventional clinical models, potentially offering a new paradigm in UTUC risk stratification and management.

A key finding in the study is the prognostic value of PRF characteristics. This aligns with recent research by Yanagi et al., who demonstrated that high PRFS was associated with significantly lower progression-free survival rates compared to low PRFS in patients with renal pelvic urothelial carcinoma without hydronephrosis [9]. The radiomics approach extends this concept by quantifying subtle texture changes in the perirenal fat, potentially capturing early signs of tumor invasion or alterations in the tumor microenvironment.

Similarly, Chung et al. found that PRFS was an independent prognostic factor for both recurrence-free survival and cancer-specific survival in patients with ureteral urothelial carcinoma [10]. They reported that patients with PRFS had significantly worse 5-year recurrence-free survival (58.1% vs. 77.9%, *p* = 0.029) and cancer-specific survival (66.4% vs. 86.8%, *p* = 0.009) compared to those without PRFS. The radiomics-based approach potentially offers a more nuanced analysis of these PRF changes, which could lead to improved prognostic accuracy.

Building upon the previous work on UTUC radiomics, which focused solely on tumor ROI and achieved a C-index of 0.74 [18], the current study demonstrates the added value of incorporating PRF radiomics. Combining features from both the tumor and perirenal fat resulted in an increase in model stability and performance, achieving a C-index of 0.784.

This improvement underscores the importance of considering the tumor microenvironment, particularly the perirenal fat, in prognostic modeling for UTUC.

The combined model achieved a C-index of 0.784, representing an improvement over several existing prognostic models for UTUC. This performance aligns with recent studies focusing on preoperative risk classification in UTUC. For instance, Somiya et al. developed a preoperative risk classification model for intravesical recurrence after laparoscopic radical nephroureterectomy, which demonstrated good discriminative ability [19]. The model’s integration of radiomics features from PRF may provide additional prognostic information not captured by conventional clinical and imaging parameters.

In the multivariate analysis, hydronephrosis emerged as a significant factor in the combined model. This finding aligns with the work of Petros, who highlighted the importance of hydronephrosis in the clinical presentation and evaluation of UTUC [20]. However, the model’s incorporation of radiomics features may provide a more comprehensive assessment of disease extent and prognosis.

The radiomics features identified in the study demonstrated strong prognostic value. While direct comparisons with other radiomics studies in UTUC are limited due to the focus on perirenal fat, these features appear to capture important aspects of the tumor microenvironment. This novel approach of analyzing PRF texture could provide additional insights into tumor behavior and progression that are not apparent through conventional imaging assessment alone.

Our findings build upon the growing body of evidence suggesting that perinephric changes, particularly PRF stranding, have significant prognostic implications in UTUC. Quantifying these changes through radiomics analysis enables detection of subtle variations that are not visually apparent but carry important prognostic information.

The findings have potential implications for clinical practice. The integration of PRF radiomics into prognostic models could enhance preoperative risk stratification, potentially influencing treatment decisions such as the use of neoadjuvant chemotherapy or the extent of lymph node dissection during surgery. Moreover, this approach could help identify patients who might benefit from more intensive follow-up protocols post-surgery.

Importantly, the PRF segmentation method used in the study can be easily applied in clinical settings. It offers a significant advantage over tumor-focused radiomics approaches as it is less sensitive to tumor delineation errors. The semi-automated nature of the method reduces the subjectivity associated with manual tumor segmentation, potentially leading to more reproducible results across different centers and observers. This ease of application and reduced sensitivity to tumor boundary definition could facilitate broader adoption of radiomics in UTUC management.

Despite these promising results, the study has several limitations. Firstly, the relatively small sample size and the distribution of tumor grades and stages within the study cohort, which may have limited the statistical power to detect significant associations with these established clinical variables. Future studies with larger, more diverse populations are needed to validate these findings.

Secondly, the retrospective, single-center design may introduce bias and limit the applicability of the findings to diverse patient populations. Future multi-center prospective studies are needed to validate these findings and assess the model’s performance across different clinical settings and patient demographics.

Additionally, while the feature selection process was rigorous, the biological significance of these radiomics features remains to be elucidated. Further research is needed to understand the underlying mechanisms linking PRF texture to UTUC prognosis.

Future research should focus on external validation of the model using larger, multi-institutional cohorts. Exploration of the biological mechanisms underlying the prognostic value of PRF radiomics in UTUC is also crucial. In addition, future research could also explore the application of advanced AI algorithms, such as deep learning and ensemble methods, to potentially improve predictive performance. These approaches may capture complex nonlinear relationships between radiomics features and patient outcomes. Integration of this approach with molecular markers and other emerging biomarkers could further enhance prognostic accuracy and personalize treatment strategies.

Lastly, while the semi-automated segmentation method offers advantages in terms of reproducibility and ease of use, further work is needed to standardize this approach across different imaging protocols and CT scanner types to ensure consistent results in varied clinical settings.

## 5. Conclusions

The study demonstrates the potential of integrating PRF radiomics with clinical data to enhance prognostic accuracy in UTUC. This novel approach not only outperforms traditional clinical models but also provides insights into the role of the tumor microenvironment in UTUC progression. As the field advances toward more personalized cancer care, integrative models could play a crucial role in improving patient outcomes by enabling more accurate risk stratification and tailored treatment strategies. The relative ease of applying PRF radiomics analysis makes it a promising tool for clinical implementation, potentially bridging the gap between advanced imaging analysis and routine clinical practice in UTUC management.

## Figures and Tables

**Table 1:** Patient characteristics and tumor features (*n* = 103). [Abbreviations: BMI, body mass index.]

Characteristic	Value
Age, median (range)	74 years (49–93)
Gender, *n* (%)	
Male	61 (59%)
Female	42 (41%)
Smoking Status, *n* (%)	
Current/Former	80 (78%)
Never	23 (22%)
BMI Category, *n* (%)	
Normal	34 (33%)
Overweight	35 (34%)
Obese	34 (33%)
Tumor Location, *n* (%)	
Renal Pelvis	49 (48%)
Ureter	54 (52%)
Histological Grade, *n* (%)	
High grade	73 (71%)
Low grade	30 (29%)
T Stage, *n* (%)	
T1	58 (56%)
T2	18 (18%)
T3 or T4	27 (26%)
Carcinoma in situ, *n* (%)	25 (23%)
Hydronephrosis, *n* (%)	25 (23%)
Multifocal, *n* (%)	38 (35%)
Tumor size, mean ± SD (cm)	1.97 ± 0.83
Deceased, *n* (%)	58 (54%)
Recurrence, *n* (%)	31 (29%)

**Table 2:** Univariate analysis results for clinical variables. [Abbreviations: CI, confidence interval; Exp. Coef., exponential of coefficients.]

Variable	Estimate	Std Error	Z Value	*p* Value	Exp (Coef)	95% CI
Tumor size	−0.41	0.25	−1.69	0.091	0.66	1.50–2.91
Size	0.10	0.18	0.56	0.57	1.10	2.19–4.75
Grade	0.15	0.31	0.49	0.62	1.16	1.89–8.41
Smoker	−0.25	0.18	−1.42	0.16	0.78	1.73–3.01
Cytology	−0.05	0.29	−0.16	0.87	0.95	1.71–5.46
Metastasis	0.29	0.53	0.55	0.58	1.34	1.61–42.74
Hydronephrosis	−0.53	0.36	−1.46	0.14	0.59	1.34–3.31
Body mass index	0.01	0.03	0.45	0.65	1.01	2.60–2.93
Stage	−0.49	0.32	−1.51	0.13	0.62	1.39–3.18
Multifocal	0.11	0.31	0.37	0.71	1.12	1.85–7.67
Location	−0.03	0.15	−0.18	0.86	0.97	2.06–3.69
Side	0.04	0.15	0.26	0.79	1.04	2.17–4.03
Gender	−0.12	0.15	−0.77	0.44	0.89	1.94–3.31
Age at operation	−0.04	0.17	−0.23	0.82	0.96	1.98–3.87

**Table 3:** Univariate analysis results for the top 15 significant radiomics features. [Abbreviations: CI, confidence interval; Exp. Coef., exponential of coefficients; Std. Error, standard error.]

Variable	Estimate	Std Error	Z Value	*p* Value	Exp (Coef)	95% CI
original_glcm_InverseVariance	−0.74	0.17	−4.35	<0.001	0.48	1.40–1.94
logarithm_firstorder_Entropy	−0.61	0.17	−3.61	<0.001	0.54	1.48–2.13
original_glszm_LargeAreaEmphasis	0.47	0.14	3.35	<0.001	1.60	3.37–8.18
exponential_glszm_GrayLevelNonUniformity	−0.69	0.20	−3.35	<0.001	0.50	0.34–0.75
wavelet.HHL_gldm_LargeDependenceLowGrayLevelEmphasis	0.65	0.20	3.21	0.0013	1.92	3.63–17.46
wavelet.HHL_glszm_LargeAreaEmphasis	0.59	0.19	3.15	0.0016	1.80	3.49–13.45
wavelet.LHL_gldm_LargeDependenceLowGrayLevelEmphasis	0.56	0.18	3.10	0.0019	1.75	3.41–11.98
original_gldm_LargeDependenceLowGrayLevelEmphasis	0.48	0.16	3.03	0.0024	1.61	3.27–8.94
wavelet.HHL_firstorder_Maximum	−0.61	0.20	−3.03	0.0025	0.54	1.44–2.24
logarithm_glszm_GrayLevelNonUniformityNormalized	0.45	0.15	2.97	0.0029	1.57	3.21–8.33
wavelet.LHH_gldm_DependenceEntropy	−0.45	0.15	−2.93	0.0034	0.64	1.61–2.37
exponential_gldm_LargeDependenceHighGrayLevelEmphasis	0.44	0.17	2.52	0.0118	1.55	3.01–8.77
lbp.2D_firstorder_InterquartileRange	−0.37	0.15	−2.49	0.0127	0.69	1.68–2.52
wavelet.HHL_glszm_GrayLevelVariance	−0.49	0.20	−2.49	0.0128	0.61	1.52–2.46
wavelet.HHL_gldm_DependenceNonUniformityNormalized	0.40	0.17	2.41	0.0158	1.49	2.94–7.90

**Table 4:** Multivariate cox regression analysis—clinical, radiomics, and combined models. [Abbreviations: CI, confidence interval; Exp. Coef., exponential of coefficients; Std. Error, standard error.]

Model	Feature	Coef	Std_Error	95% CI	*p*_Value
Clinical	Size	1.02	0.19	0.70–1.49	0.91
	Grade	2.02	0.44	0.85–4.79	0.11
	Smoker	0.72	0.19	0.49–1.05	0.09
	Cytology	0.56	0.39	0.26–1.21	0.14
	Stage	0.38	0.43	0.16–0.88	**0.02**
	Hydronephrosis	0.45	0.40	0.20–0.98	**0.04**
PRF radiomics	wavelet.LHH_gldm_DependenceEntropy	0.40	0.22	0.26–0.61	**<0.001**
	exponential_glszm_GrayLevelNonUniformity	0.49	0.19	0.33–0.71	**<0.001**
	exponential_gldm_LargeDependenceHighGrayLevelEmphasis	1.57	0.18	1.12–2.22	**0.01**
Combined PRF radiomics + clinical	Stage	0.46	0.35	0.23–0.92	**0.03**
	Hydronephrosis	0.37	0.39	0.17–0.80	**0.01**
	wavelet.LHH_gldm_DependenceEntropy	0.38	0.22	0.24–0.58	**<0.001**
	exponential_glszm_GrayLevelNonUniformity	0.37	0.21	0.25–0.57	**<0.001**
	exponential_gldm_LargeDependenceHighGrayLevelEmphasis	1.64	0.19	1.14–2.38	**0.01**
Combined PRF + Tumor radiomics + clinical	Stage	0.45	0.36	0.22–0.91	**0.03**
	Hydronephrosis	0.47	0.43	0.20–1.11	0.08
	wavelet.LHH_gldm_DependenceEntropy	0.44	0.25	0.27–0.72	**<0.001**
	exponential_gldm_LargeDependenceHighGrayLevelEmphasis	1.51	0.19	1.04–2.21	**0.03**
	exponential_glszm_GrayLevelNonUniformity	0.39	0.24	0.24–0.63	**<0.001**
	T.wavelet.LHL_glcm_Correlation	1.24	0.20	0.84–1.84	0.28
	T.wavelet.LLH_glcm_InverseVariance	1.40	0.24	0.88–2.25	0.16

**Table 5:** Comprehensive performance metrics for clinical, radiomics, and combined models.

Model	C-Index (95% CI)	Integrated Brier Score	Optimism-Corrected C-Index	AUC at 12 Months	AUC at 36 Months	AUC at 60 Months
Clinical	0.65 (0.55–0.76)	0.19	0.63	0.53	0.75	0.69
Radiomics	0.76 (0.68–0.84)	0.14	0.73	0.93	0.75	0.80
Combined	0.78 (0.71–0.86)	0.13	0.75	0.89	0.79	0.84

## Data Availability

All relevant data are included within the manuscript. For further details, the corresponding author (A.A.M.) may be contacted upon reasonable request.

## Supplementary Materials

The following supporting information can be downloaded at: https://www.mdpi.com/article/10.3390/cancers16223772/s1, Figure S1. Contrast-enhanced CT urogram of a patient with renal pelvis upper tract urothelial carcinoma (UTUC); Figure S2. Contrast-enhanced CT urogram of a patient with ureteral UTUC; Table S1: Table: Definitions and Explanations of Key Radiomics Features Associated with Survival Outcomes.
