# Supplementary material for: Perirenal Fat CT Radiomics-Based Survival Model for Upper Tract Urothelial Carcinoma: Integrating Texture Features with Clinical Predictors
Source: Cancers (Basel). 2024 Nov 8;16(22):3772. doi: 10.3390/cancers16223772 (PMC11593147; doi:10.3390/cancers16223772)
Supplement: Supplementary file 1 [file cancers-16-03772-s001.zip › cancers-3249323-supplementary.pdf]

Supplementary

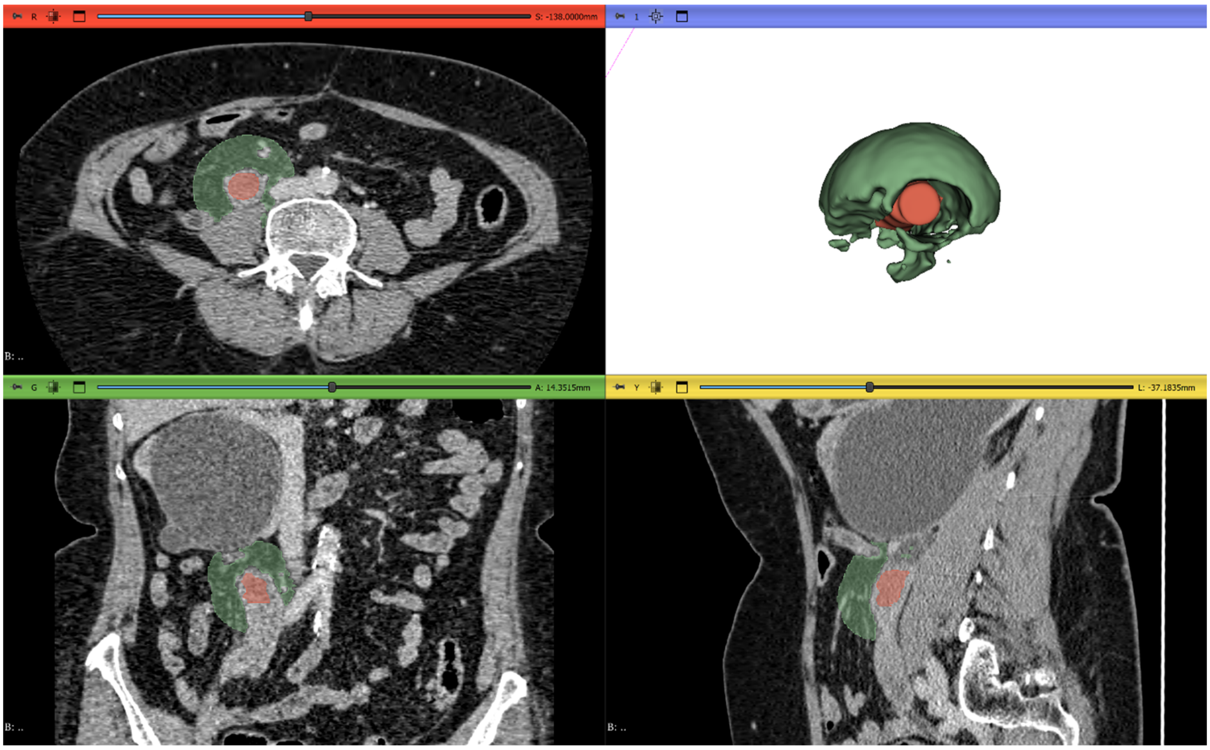

Figure S1. Contrast-enhanced CT urogram of a patient with renal pelvis upper tract urothelial carcinoma (UTUC). The tumour is segmented and highlighted in red, while the perirenal fat is segmented in green using the semi-automated method. Additionally, a 3D graphical representation of the segmented volumes is presented.

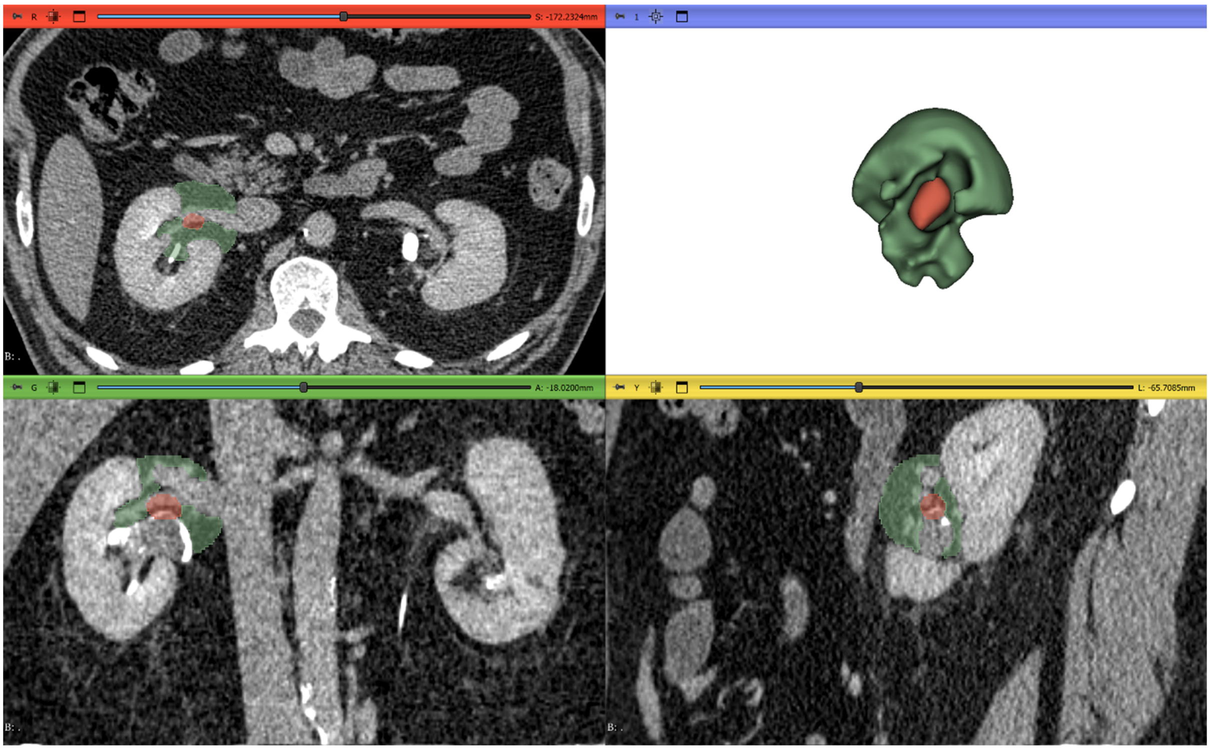

Figure S2. Contrast-enhanced CT urogram of a patient with ureteral UTUC. The tumour segmentation is displayed in red, and the perirenal fat segmentation is shown in green, generated by the semi-automated method.

Table S1: Table: Definitions and Explanations of Key Radiomics Features Associated with Survival Outcomes

Table: Definitions and Explanations of Radiomics Features

Radiomics Feature	***Definition***	***Explanation***
Original GLCM Inverse Variance	Measures local homogeneity using GLCM.	Higher values indicate more uniform textures; lower values suggest greater texture complexity.
Logarithm First-Order Entropy	Entropy of pixel intensities after logarithmic transformation.	Higher entropy reflects greater randomness or heterogeneity in the image.
Original GLSZM Large Area Emphasis	Emphasizes large homogeneous zones using GLSZM.	Higher values indicate larger uniform regions within the tissue.
Exponential GLSZM Gray Level Non-Uniformity	Assesses gray-level variability across zones after exponential transformation.	Higher values suggest greater intensity variability, indicating tissue heterogeneity.

Wavelet HHL GLDM Large Dependence Low Gray Level Emphasis	Highlights large dependencies among low-intensity pixels in high-frequency components.	Captures extensive low-intensity areas, possibly related to fatty infiltration or edema.
Wavelet HHL GLSZM Large Area Emphasis	Measures large homogeneous zones in specific frequency bands after wavelet transform.	Identifies texture patterns at different scales, revealing subtle structural changes.
Wavelet LHL GLDM Large Dependence Low Gray Level Emphasis	Highlights large dependencies among low-intensity pixels in other frequency components.	Indicates extensive low-intensity regions, possibly correlating with fibrosis or necrosis.
Original GLDM Large Dependence Low Gray Level Emphasis	Measures large dependencies among low-intensity pixels in the original image using GLDM.	Suggests uniform low-density regions, such as fat or fluid accumulation in perirenal fat tissue.
Wavelet HHL First-Order Maximum	Maximum intensity value after high-frequency wavelet transform.	Represents the brightest regions in specific frequency bands, potentially indicating calcifications or dense structures.
Logarithm GLSZM Gray Level Non-Uniformity Normalized	Normalized gray-level variability across zones after logarithmic transformation.	Higher values indicate greater heterogeneity in gray-level intensities within zones.
Wavelet LHH GLDM Dependence Entropy	Entropy of pixel dependencies after wavelet transform.	Higher values reflect complex textures and irregular patterns, associated with tissue heterogeneity.
Exponential GLDM Large Dependence High Gray Level Emphasis	Emphasizes large dependencies among high-intensity pixels after exponential transform.	Higher values indicate extensive areas of high-intensity dependence, possibly related to dense or fibrotic tissue associated with aggressive tumors.
LBP 2D First-Order Interquartile Range	Interquartile range of intensities after LBP transformation.	Reflects variability in local texture patterns; higher values suggest greater heterogeneity.
Wavelet HHL GLSZM Gray Level Variance	Variance of gray-level intensities in zones after wavelet transform.	Higher variance indicates wide intensity ranges within zones, reflecting tissue heterogeneity.
Wavelet HHL GLDM Dependence Non-Uniformity Normalized	Normalized non-uniformity of pixel dependencies after wavelet transform.	Higher values indicate less uniform dependencies, signifying complex and irregular textures.

Note: These features are derived from advanced image analysis techniques applied to CT images of perirenal fat. They capture subtle textural and intensity variations not visible in standard imaging but significant for prognostic assessment in UTUC.

- **Wavelet LHH GLDM Dependence Entropy** and **exponential glszm Gray Level Non-Uniformity** were significantly associated with better survival outcomes ($p < 0.001$). Higher values of these features indicate greater complexity and heterogeneity in the perirenal fat tissue, which may reflect a favourable tumour microenvironment or effective host response.
- **Exponential GLDM Large Dependence High Gray Level Emphasis** was associated with poorer survival outcomes. Higher values suggest the presence of large, homogeneous regions of high-intensity pixels, possibly indicating pathological changes such as fibrosis or dense tissue infiltration associated with aggressive disease.
